# Can farmers’ climate change adaptation strategies ensure their food security? Evidence from Ethiopia

**DOI:** 10.1080/03031853.2023.2230959

**Published:** 2023-07-05

**Authors:** Girma Gezimu Gebre, Yuichiro Amekawa, Aneteneh Ashebir

**Affiliations:** a The Japan Society for the Promotion of Science (JSPS) Postdoctoral Research Fellowship Program, Ritsumeikan University, Kyoto, Japan; b Department of Agribusiness and Value Chain Management, Faculty of Environment, Gender and Development Studies, Hawassa University, Hawassa, Ethiopia; c College of International Relations, Ritsumeikan University, Kyoto, Japan

**Keywords:** Adaptation, climate change, food security, propensity score matching, Ethiopia

## Abstract

Climate change poses a significant threat to the sustainability of agricultural production among smallholder farm households in Ethiopia. To reduce the adverse effects of climate risks, farm households have sought to adopt different adaptation strategies. This study investigates factors influencing farm households’ choice of climate adaptation strategies and associated effects on their food security in Ethiopia using data collected from 516 farm households from three regions. A multivariate probit and propensity score matching models were used to analyze data. Major adaptation strategies adopted by the farm households in the study area are planting drought-tolerant crop varieties (60%), changing the planting dates (53%), growing diversified crops (49%), and diversifying the sources of household income (45%). Results suggest that older farm household heads are more likely to use drought-tolerant crop varieties to reduce climate risks. Farm households with larger farmland size and those with more years of experience in farming are more likely to use drought-tolerant crop varieties and crop diversification strategies. Farm households with larger family size are more likely to use crop and income diversification strategies and change the planting dates against the backdrop of a high risk of climatic shocks. Membership in input supply cooperatives, frequency of contact with extension agents, and access to information on expected rainfall and temperature are positively associated with different adaptation practices adopted by farm households. Farm households who have adopted climate adaptation strategies have higher food security status (by 2.3–2.8%) compared to those who have not. Thus, the farm households’ climate adaptation practices have positive food security effects in Ethiopia.

## 1. Introduction

Climate change poses a significant growing threat to the productivity of their agricultural systems (Teklewold et al. 2013; Ajilogba and Walker 2021) and associated food security (Inter-governmental Panel on Climate Change [IPCC], 2014; Haq et al. 2021; Shahbaz et al. 2021). It challenges the 2030 agenda of Sustainable Development (IPCC 2022), which was adopted by the United Nations General Assembly on September 25, 2015, with an objective to “*end hunger and ensure access by all people to safe, nutritious and sufficient food all year round”* (Food and Agriculture Organization (FAO) 2017). Climate risks disproportionately affect vulnerable populations living in agricultural communities in developing countries (Di Falco et al. 2012; Rahut and Ali 2017; Amole and Ayantunde 2019; Teklewold et al. 2019a). Climate risk is expected to affect many more people in more areas in the future (De Pinto et al. 2019). The worst-hit areas will be underdeveloped economic regions of the world, including Sub-Saharan Africa (SSA) (Akinnagbe and Irohibe 2014; Hadebe et al. 2016; Mekonnen et al. 2020; Zakari et al. 2022), where food security is already a big challenge and human populations are highly vulnerable to climatic and other shocks (Di Falco et al. 2011; Drammeh et al. 2019; Kabubo-Mariara and Mulwa 2019; Filho et al. 2020; 2021; Ayal et al. 2021; Ndiritu and Muricho 2021; Gebre and Rahut 2021).

The adverse effects of climate risks in East African countries are very severe due to the interaction of multiple factors, including high population growth, over dependence on rain-fed agriculture, poor availability and quality of meteorological data, extreme poverty, and knowledge gaps (IPCC 2014; Agidew and Singh 2018; Drammeh et al. 2019; Kabubo-Mariara and Mulwa 2019; Teklewold et al. 2019b; Aryal et al. 2021; Gebre and Rahut 2021). Climate risk-adapted development can enable East African countries to diversify their livelihood sources and become less reliant on sectors that are more vulnerable to climate risks, while helping to strengthen their capacity to mitigate adverse effects of climate risk (World Bank 2010). Early adaptation actions could promote development by reducing risks and costs associated with asset losses from climate-related disasters, reducing infrastructure repair costs, and creating new opportunities (World Bank 2019).

Ethiopia has been identified as one of the East African countries that are most vulnerable to climate risks and is frequently faced with climate-related hazards, commonly drought and floods (Belay et al. 2017; Teklewold et al. 2019a; 2019b; Gezie 2019) which have significant impacts on agricultural productivity and associated food security (Alemu and Mengistu 2019). In recognition of this, the Government of Ethiopia has developed adaptation and mitigation strategies to address climate risks. Most of these strategies focus on efforts to enhance the adoption of climate-smart agriculture in agricultural production. Importantly, the adoption of climate-smart agricultural practices and associated technologies has been shown to improve the quality of the food consumed in terms of increased calorie and protein availability, leading to improved nutritional outcomes (Stifel and Minten 2017; Teklewold et al. 2019a; International Institute for Sustainable Development 2022). Ethiopia is also one of the few countries that have developed a National Adaptation Plan (NAP) to reduce vulnerability to the impacts of climate risks by building adaptive capacity and resilience (Federal Democratic Republic of Ethiopia 2020).

Numerous studies are now available on factors affecting the adoption of climate risk adaptation strategies in various developing countries (e.g., Di Falco et al. 2012; Belay et al. 2017; Brüssow et al. 2017; 2019; Hansen et al. 2019; Teklewold et al. 2019b; Hirpha et al. 2020; Issahaku et al. 2021; Mihiretu et al., 2021; Shahbaz et al. 2022). However, studies on the effectiveness of adopted strategies for the food security of rural households are scarce in developing countries, including in Ethiopia. This paper aims to contribute to the literature on climate change and agriculture (e.g., Di Falco et al. 2011; Teklewold et al. 2019a; Gebre and Rahut 2021) by providing a micro-perspective of people’s adaptation to climate risk and its effects on food security in Ethiopia. Specifically, the paper investigates the determinants in the choice of multiple climate risk adaptation strategies by smallholder farm households using multivariate probit regression. It also assesses the impact of the use of these adaptation strategies on the food security of households using a Propensity Score Matching method.

The rest of the paper is structured as follows: section two discusses the conceptual framework and analytical methods; section three describes the study area, data, and sampling procedures; section four presents the results and discussion; and section five concludes the study with a note on policy implications.

## 2. Conceptual framework and analytical methods

### 2.1 Conceptual framework

Climate risks involve the possibility of the occurrence of extreme weather events and associated natural hazards, such as erratic rainfall, intra-seasonal dry spells, high temperatures, frequent drought, land degradation, cyclones, floods, and soil erosion. These climate adversities can negatively affect agricultural production (crop and livestock), and hence, the food security of farm households (Di Falco et al. 2011; IPCC 2014; Ali and Erenstein 2017; Eitzinger et al. 2018; Kabubo-Mariara and Mulwa 2019; Teklewold et al. 2019b; Gebre and Rahut 2021; Ndiritu and Muricho 2021; Gebre et al. 2023a; Gebre et al. 2023b). To reduce the adverse effects of climate risks on their food security, farm households in developing countries adopt various adaptation strategies (Amare and Simane 2017; Aryal et al. 2020; Gebre et al. 2023a; Gebre et al. 2023b). Farm households’ adoption (choice) of adaptation strategies against climate risks depends on multiple factors, including household demography, socioeconomic characteristics, as well as other institutional and biophysical factors (Aryal et al. 2020; 2021; Atube et al. 2021; Mairura et al. 2021; Gebre and Rahut 2021). If the climate adaptation strategies adopted by farm households are adequate and effective, it will increase their agricultural production and better ensure their food security, and vice versa. Income diversification adaptation strategies can also positively influence their food security status by improving their financial capital base to purchase foods (Di Falco et al. 2012; Zakari et al. 2022).

### 2.2 Analytical framework

In Ethiopia, farm households adopt different adaptation strategies to mitigate climate risks and ensure food security. Therefore, we first employed a multivariate probit model to identify the choice determinants of multiple climate adaptation strategies, including the use of drought-tolerant crop varieties, crop diversification, changing the planting dates, and income diversification. Employing other qualitative choice models, such as univariate probit and logit, is not appropriate in this case as they may generate biased coefficients. Univariate probit and logit models are based on the assumption of the independence of error terms pertaining to different adaptation practices applied by farm households (Greene 2019). Possible complementarities could also occur between various adaptation strategies used by farmers (Greene 2019). In Ethiopia, farm households are more likely to adopt multiple adaptation strategies simultaneously in order to reduce climate risk related to their food security (Teklewold et al. 2019a; 2019b). Using a multivariate probit model in this condition yields unbiased coefficients (Wooldridge 2012; Greene 2019).

Given a set of adaptation strategies, we assume that a risk-averse farm household (S) will choose an adaptation strategy $\left({\text{F}}_{\text{i}1}\right)$ that yields a higher utility (Y) relative to the alternative adaptation strategy $\left({\text{F}}_{\text{i}2}\right)$, as shown in Eq. (1):

(1) $$U[S(Y)]=U\left[S\left({\text{F}}_{i1}\right)\right]>U\left[S\left({\text{F}}_{i2}\right)\right]$$

Since the utility could not be observed, it is represented as a function of observable components as expressed in Eq. (2):

(2) $$Y_{rf}^{*}=\alpha_{f}X_{rf}+\beta_{f}V_{rf}+\varepsilon_{f}\;where(f=1,\ldots .m)$$

$${\text{Y}}_{\text{rf}}=1\;\text{if}\;{\text{Y}}_{\text{rf}}^{*}>0\;\text{and}\;0\;\text{if otherwise}$$

where $Y_{If}^{*}$ represents the latent variable indicating the unobserved outcome, and it is associated with $f^{th}$ which represents climate change adaptation strategies. The $Y_{rf}$ denotes the binary dependent variable, and (f=1,…n) represents the strategies adopted by farm households in the study area (i.e., use of drought-tolerant crops varieties, crop diversification, changing the planting dates, and income diversification). The farm household is assigned a value of 1 if any adaptation strategy was chosen, and 0 if otherwise. $X_{rf}$ is the vector of the explanatory variables in the model. $\alpha_{f}$ and $\beta_{f}$ represent the parameters to be estimated. The error term $\varepsilon_{f}$ in the model have multivariate normal distributions, with zero (representing a conditional mean) and a unit variance.

Second, a propensity score matching (PSM) method was employed to estimate the effect of adaptation strategies on farm households’ food security status in Ethiopia. The expected treatment effect for the treated population is of primary significance, and it is given as

(3) $$ATT=\text{E}(\text{Δ}|\text{D}=1)=E\left(Y_{1}|x,D=1\right)-E\left(Y_{0}|x,D=1\right)$$

where ATT represents the average treatment effect for the treated, $Y_{1}$ represents the value of the outcome for adopters of a climate risk adaptation strategy, and $Y_{0}$ denotes the value of the same explanatory variable x for non-adopters of the climate risk adaptation strategy .^1^ As noted above, the major problem with this procedure is that the counterfactual $E\left(Y_{0}|x,D=1\right)$ is not based on empirical observation. Although the value of ATT (the difference between $E\left(Y_{1}|x,D=1\right)-E\left(Y_{0}|x,D=0\right))$ can be estimated, it is potentially a biased estimator (coefficient). In the absence of experimental data, the PSM can be applied to account for this sample selection bias due to counterfactual effects (Dehejia and Wahba 2002). To create the condition of a randomized experiment, the PSM applies the conditional independence assumption, which implies that once Z is controlled for, a climate risk adaptation strategy is random and uncorrelated with the outcome variables (food security in the case of this study). That is, in short, the outcomes are independent of treatment. The PSM can be expressed as:

(4) P(Z)=Pr{D=1∣Z}=E{D∣Z}

where D is the indicator for adoption and Z is the vector of pre-adoption characteristics (Abara and Singh 1993). The conditional distribution of Z given P(Z) is similar between the adopter and non-adopter groups. After estimating the propensity scores, the average treatment effect for the treated (ATT) can be estimated as:

(5) $$ATT=E\left\{Y_{1}-Y_{0}|x,D=1\right\}=E\left\{E\left\{Y_{1}-Y_{0}|x,D=1,p(Z)\right\}\right\}\;=E\left\{E\left\{Y_{1}|x,D=1,p(Z)\right\}-E\left\{Y_{0}|x,D=0,p(Z)\right\}|x,D=0\right\}$$

Several techniques have been developed to match non-adopters with adopters of similar propensity scores. The PSM depends on the conditional independence (see, Caliendo and Kopeinig 2008) and the common support condition (see, Bryson et al. 2002) assumptions. The most important variable of interest for the PSM is ATT. In our study’s context, ATT is the difference in the outcome of farm households having used climate risk adaptation strategies and similar farm households not adopting it. In PSM estimation, it is important to determine the region of common support to check the overlap in the propensity score distribution between the adopter and non-adopter groups. Applying matching algorithms helps us to choose and determine the region of common support in a PSM analysis. Therefore, we employed two types of PSM algorithms commonly used in PSM analysis to check the level of diversity in the obtained results. The nearest neighbor matching (NNM) and kernel-based matching (KBM) algorithms were used. After matching for NNM and KBM, several balancing tests were employed to assess the matching quality, such as checking a reduction in the median absolute bias, the value of R^2, and the p-value of joint significance of covariates before and after matchings (Becker and Ichino 2002; Caliendo and Kopeinig 2008; Ali and Erenstein 2017; Rahut and Ali 2018; Gebre et al. 2023a).

The food security measure/cut-off point was calculated using the Household Food Insecurity Access Prevalence indicators (Coates et al. 2007; Headey and Ecker 2012). For each farm household, the Household Food Insecurity Access category variable was calculated using the assigned codes of the degree of food security into which it fell (see Appendix). Accordingly, based on their severe responses, four sequential categories of food security states were created: food-secure, mildly food-insecure, moderately food-insecure, and severely food-insecure. Each category was calculated by dividing the number of farm households in one category by the total number of farm households in the four categories. Due to the small sample size, all three food-insecure statuses (mildly, moderately, and severely) were merged into “food-insecure” and the rest into “food-secure” categories. Thus, the dependent variable (outcome variable) was binary, with “one” assigned to a food-secure household and “zero” to a food-insecure household.

## 3. Study area, data, and sampling procedures

### 3.1 Study area

**Figure 1.**
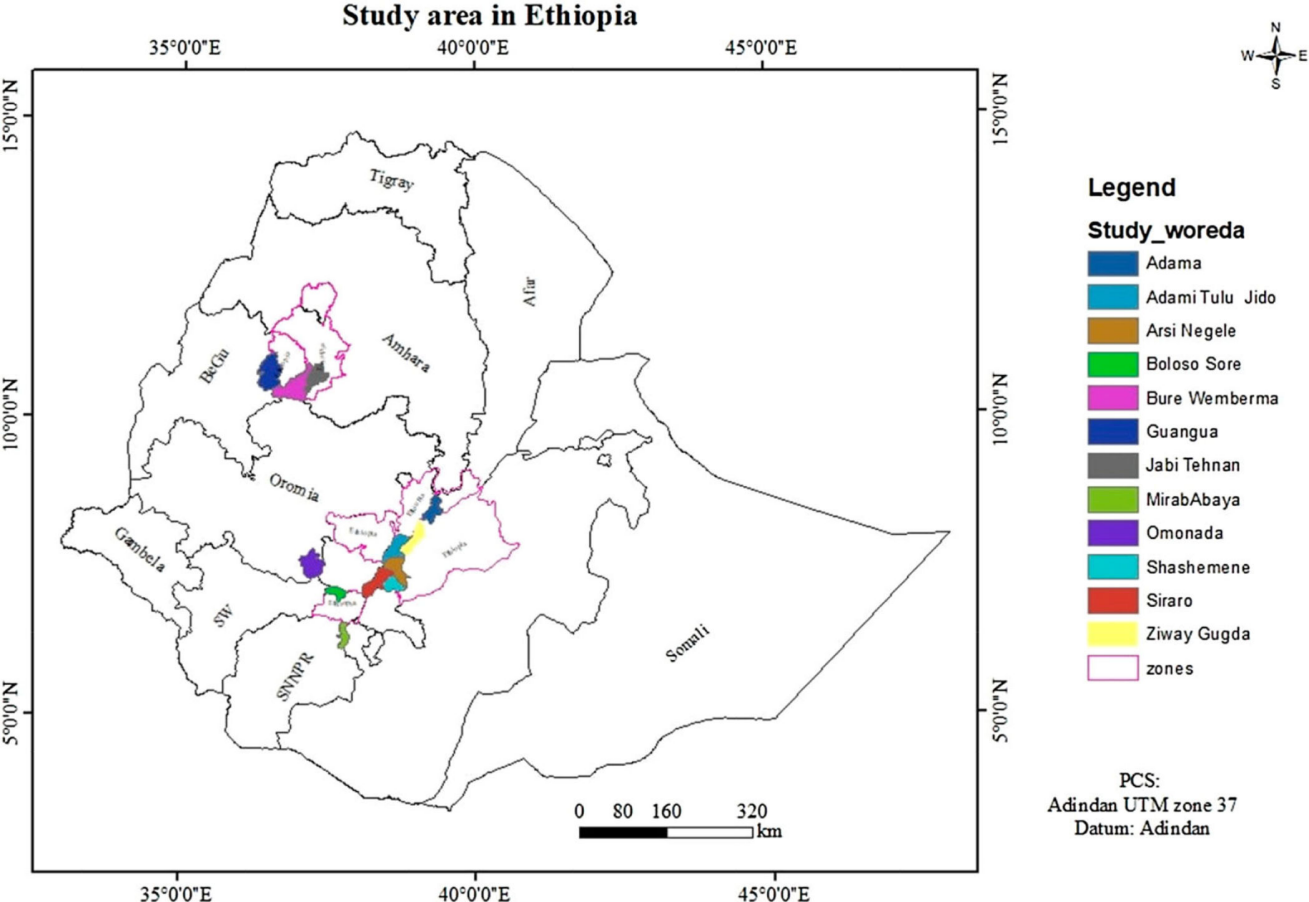
Map of the study area. Source: authors.

The study is based on a set of household survey data collected in December 2018 through the Stress Tolerant Maize for Africa (STMA) project. The STMA project aimed to help smallholder farmers mitigate the combined effects of multiple stressors, such as drought, heat, poor soil fertility and diseases, that affect their maize farming. Accordingly, it also aimed to improve their food security and livelihoods. The sampling procedure to identify the study areas and respondent households was designed by researchers from the International Wheat and Maize Research Center (CIMMYT) in collaboration with agricultural personnel of regional and district-level governments in Ethiopia. The survey area involved 12 districts (woredas) across three regions, including Amhara region (Guangua, Bure Wemberma, and Jabi Tehnan districts), Oromia region (Adama, Adami Tulu, Arsi Negele, Omonada, Shashemene, Siraro, and Zeway Dugda, districts), and the South Nation, Nationalities, and People region (Mirab Abaya and Boloso Sore districts) (see Figure 1). The identification of the districts was based on their potential for the production of major crops in the country. The major crops in the districts are maize, teff, haricot bean, wheat, sorghum, pepper, and finger millet.

### 3.2 Data and sampling procedures

Respondents, districts and sub-districts (kebele) were identified for the survey by means of a multistage sampling procedure that involved a combination of purposive and random sampling. The major crop producing districts and sub-districts (kebeles) were purposely identified on the basis of their current production potential and status. Proportional to size, the random sampling procedure was used to select, on average, two kebeles per district, where 18–20 farm households per kebele were selected from a complete household list provided by local authorities. A total of 516 households were randomly selected and interviewed in 2018. A semi-structured questionnaire was designed and used to capture a range of information related to farm household demographic and socioeconomic characteristics, and agronomic features and food security. The questionnaire also captured some individual and household characteristics, as well as institutional arrangements besetting households on farm management. Trained and experienced enumerators administered the questionnaire under the close supervision of researchers from CIMMYT.

### 3.3 Description of the variables

Table 1 presents the definition, types, and mean values of the variables included in the econometric model estimations. Farm households have used several measures to adapt to climate change in the study area, which can be classified into four major strategies for the analysis. The most dominant strategy adopted by the surveyed farm households was the use of drought-tolerant crop varieties (60%), followed by changing the planting dates (53%), crop diversification (49%), and income diversification (45%). The study disaggregated surveyed households into adopters and non-adopters of climate risk adaptation strategies and examined the impact of climate risk adaptation strategies in the food security status of the surveyed households. Of the total surveyed farm households, about 74% adopted at least one adaptation strategy, while the rest (26%) did not adopt any measures to cope with climate-change related risks.

## 4. Results and discussion

### 4.1 Descriptive results

Table 2 presents a summary of descriptive statistics by adopter and non-adopter farm households. Regarding the food security status of the surveyed households, about 60% were in the food-secure category, while the remaining 40% were in the food-insecure category. In the food-insecure category (out of the 40%), about 11% were mildly food-secure, while the rest 22% and 7% were moderately and severely food-insecure, respectively. It is also noticed that adopters were more food-secure (66%) compared to non-adopters (52%), with a significant difference at 1% level. Non-adopters were more food-insecure (48% i.e., sum of mildly (9%), moderately (26%), and severely (13%)) compared to the adopters (34% i.e., sum of mildly (12%), moderately (18%), and severely (4%)). The differences in all food-insecure categories were statistically significant. These results support the hypothesis that agricultural households that adopt climate risk adaptation strategies are more food-secure than those that do not adopt them (e.g., see Di Falco et al. 2011; Teklewold et al. 2019a). The present study results were rigorously tested using an econometric model.

Most surveyed households (91%) were headed by males, whereas females headed only about 9%. The average age of the head of the surveyed households was 48.66 years, with 31.20 years of farming experience and 5.20 years of education. The average number of household members was 6.88. The majority of the farm households in the study area were smallholders with a total average farmland size of 1.92 ha. The average farmland size was higher among adopters, while it was lower in non-adopter households. The number of extension visits was on average 2.58 for the cropping season. Distance to the main market and agricultural development agent was 8.07 and 3.05 km, respectively. Approximately, 40% of the surveyed farm household had membership in agricultural input supply cooperatives. Approximately, 61% of the surveyed farm households regularly receive information on expected rainfall and temperature. Access to climate information was significantly higher among adopters compared to non-adopter groups. A study by Di Falco et al. (2012) found a significant result on the positive relationship between access to information on future climate changes and the adoption of adaptation strategies in Ethiopia.

### 4.2 Econometric results

#### 4.2.1 Determinants for the choice of climate risk adaptation strategies

Table 3 presents the results of the multivariate probit estimation of the determinants of the farm household’ climate change adaptation strategies, including the use of drought-tolerant crop varieties, crop diversification, changing the planting dates, and income diversification. These four dependent variables are assumed to be mutually inclusive, which means a farmer could use a combination of more than one climate risk adaptation strategy in the study area. Previous studies by Teklewold et al. (2013; 2019a) found a strong complementarity among climate adaptation practices in Ethiopia. Thus, a multivariate probit model is suitable to estimate climate adaptation strategies in this study. A set of independent variables are included in the multivariate probit model based a review of relevant literature. The result of the Wald test statistics (χ^2^ = 147.60, *P* > 0.000) indicates that a set of explanatory variables included in the model significantly influences the response variables. The result of the likelihood ratio test χ^2^ (6) = 167.561 and Prob > χ^2^ = 0.000 of the independence of the error terms in the different equations indicates that the null hypothesis is rejected. Therefore, the study accepts an alternative hypothesis of independence among the different adaptation strategies, justifying the use of the multivariate probit model used in the analysis of farm households’ adoption of climate risk adaptation strategies.

The age of the household head is positively associated with growing drought-tolerant crop varieties. Similar results of a positive association between age and climate adaptation strategies were reported by Maddison (2006) and Ishaya and Abaje (2008). However, Denkyirah et al. (2016) and Ojo et al. (2021) reported a negative association between age and the adoption of climate change adaptation strategies. According to Ndiritu et al. (2014), aging can be associated with more loss of physical energy and more risk-averse tendency. Hence, the positive association between age and growing drought-tolerant crop varieties may be related to older farmers’ higher preference of risk aversion against droughts and a delay in the arrival of the rainy season in Ethiopia. The results also indicate that farm households with more experienced heads are more likely to grow drought-tolerant and diversified crop varieties. Similar findings were reported by Aryal et al. (2020), who noted that more experienced farmers have more knowledge and skills to use different adaptation strategies. However, Ado et al. (2019) and Ojo et al. (2021) reported a negative relationship between experience and climate risk adaptation strategies. Family size is positively associated with crop diversification, income diversification, and changing the planting dates. A significant positive coefficient of household size indicates that a farm household with more family members adopts more adaptation strategies to minimize climate risks. Other studies reported similar results (e.g., Atinkut and Mebrat 2016; Gautam and Andersen 2016; Kabubo-Mariara and Mulwa 2019; Megersa et al. 2022; Zakari et al. 2022).

Land is positively associated with growing drought-tolerant crop varieties and crop diversification (Table 3). This result is consistent with the generally reported positive association between farm size and technology adoption (Bryan et al. 2013; Abid et al. 2015; Gebre et al. 2019), coupled with that between farm size and adoption of climate risk adaptation strategies (Ali and Erenstein 2017; Kabubo-Mariara and Mulwa 2019; Jamshidi et al. 2020). Since land is a major proxy for the household possession of wealth, farmers with larger landholdings tend to adopt more climate risk adaptation strategies because of their financial ability to invest in new technologies and farming methods to adapt to climate risk.

The frequency of extension contact is positively associated with income diversification and changing the planting dates. This indicates the critical importance for farm households to access relevant information and other resources through extension agents in the study area when seeking to use the listed climate risk mitigating strategies. This finding complies with Di Falco et al. (2012) who confirmed that agricultural extension for climate risk adaptation, whether it be formal extension or farmer-to-farmer extension, was positively and significantly correlated to the adoption decision in Ethiopia. Households with membership in an agricultural input supply cooperative are more likely to engage in the growing of drought-tolerant crop varieties, crop diversification, income diversification, and the adjusting of the planting dates. This result is in line with findings by Aryal et al. (2020). The households with access to information on expected rainfall and temperature are more likely to engage in the planting of drought-tolerant crop varieties, crop diversification, income diversification, and the adjusting of the planting dates. Di Falco et al. (2011; 2012) found similar results that farmers in Ethiopia who were regularly informed about climate conditions were more likely to adapt to climate risk. Similar study results were reported by Nhemachena et al. (2014), Asrat and Simane (2018), Mihiretu et al. (2019), and Zakari et al. (2022).

#### 4.2.2 Impact of climate risk adaptation strategies on food security

Table 4 presents the impact of climate risk adaptation strategies on farm households’ food security based on the PSM analysis. Both nearest neighbor matching and kernel-based matching reveals that farm households that adopted a climate risk adaptation strategy have a higher food security status (2.3–2.8%) compared to those who have not adopted. This result is supported by previous studies by Di Falco et al. (2011), Ali and Erenstein (2017), Amare and Simane (2017), Ogundeji (2022), and Zakari et al. (2022), who concur that an improved adoption and use of climate risk adaptation strategies is essential for ensuring household food security.

Table 5 presents the balancing tests for the PSM. Before matching, the bias was very high (19.5%), but it was reduced to 6%–6.5% after matching. The percentage bias reduction is 69.23–72.01%. The value of R^2^ was high before matching (0.150), but it became very low after matching (0.018 for NNM and 0.017 for KBM), signifying that, after matching, both groups are very similar to each other. The *p*-value of joint significance of covariates indicates that before matching, there were systematic differences between the adopters and non-adopters, but after the adopters and non-adopters became very similar to each other after matching.

Table 6 presents the distribution of estimated propensity scores. The region of common support is [0.200, 0.986], which indicates the balancing property is satisfied.

**Figure 2.**
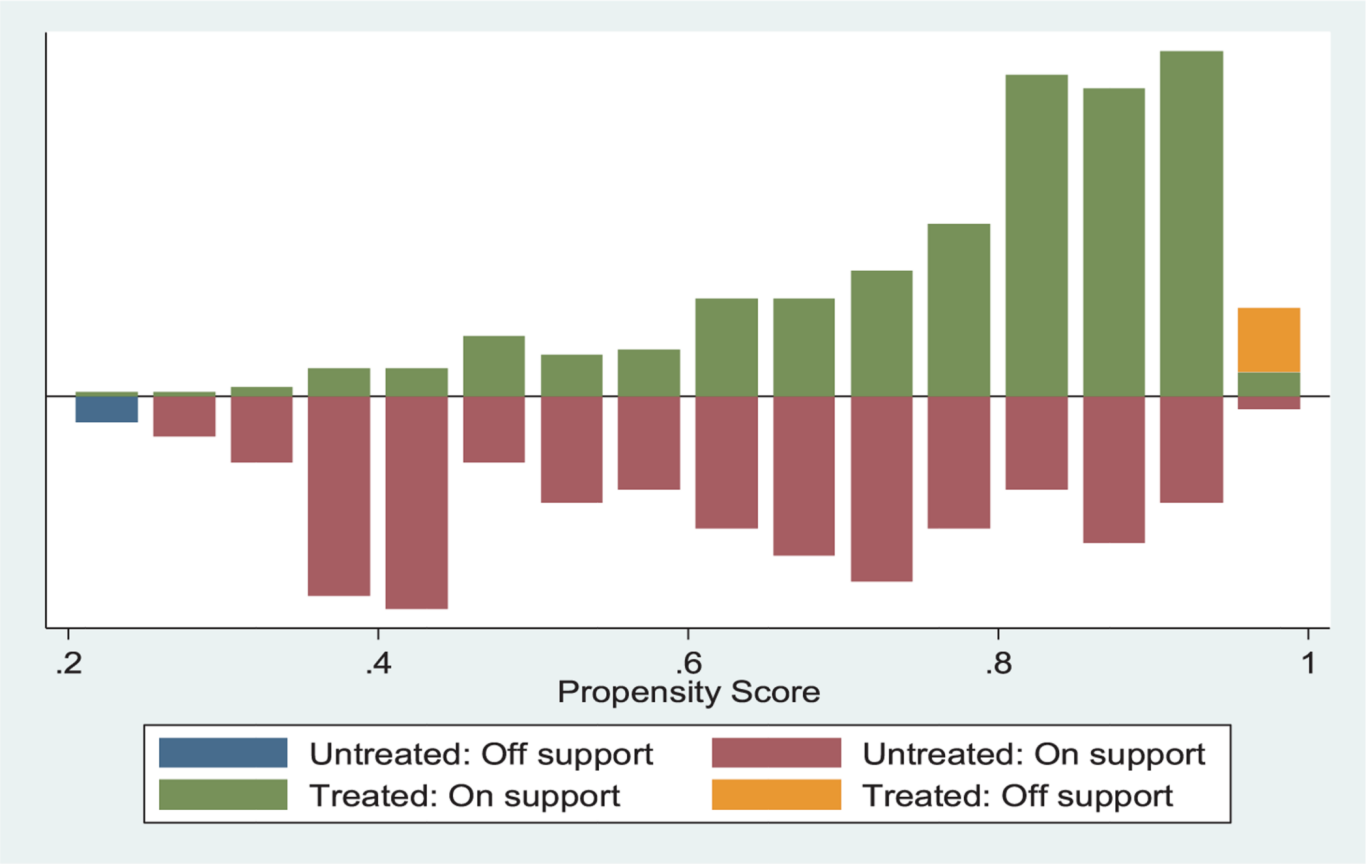
Propensity score matching estimates.

Figure 2 shows the distribution of propensity scores of matched and unmatched individuals in both groups. The result guarantees a sufficient overlap in the distribution of the propensity score between adopters and non-adopters.

## 5. Conclusion and policy implications

We examined the factors affecting farm households’ choice of climate risk adaptation strategies and associated effects on their food security in Ethiopia. Farm households in Ethiopia are using different adaptation strategies to minimize the negative impacts of climate risks. Our survey with 516 farm households from across three regions of Ethiopia in 2018 found that approximately 60% of the farm households adopted the planting of drought-tolerant crop varieties, approximately 53% changing the planting dates, approximately 49% a crop diversification strategy, and approximately 45% an income diversification strategy.

The results of the multivariate probit model suggest that older farm household heads are more likely to use drought-tolerant crop varieties to reduce climate risks. Farm households with larger farmland sizes and those with more years of experience in farming are more likely to use drought-tolerant crop varieties and crop diversification strategies. Farm households with larger family sizes are more likely to use crop and income diversification strategies and change the planting dates against the backdrop of a high risk of climatic shocks. Membership in input supply cooperatives, frequency of contact with extension agents, and access to information on expected rainfall and temperature are positively associated with different adaptation practices adopted by farm households in the survey area. More access to extension services and information would be critical in improving farmers’ knowledge and skills for adopting new agricultural technologies and practices related to climate risk adaptation in the study area.

The results of the multivariate probit estimation revealed some interesting patterns, which are unique in the context of Ethiopia, with significant policy implications. Firstly, the results highlight the importance of farm households’ knowledge and awareness about the local context, climate risk adaptation strategies, and their benefits. Secondly, the results point to the importance of wealth (e.g., farmland) regarding the ability of farmers to invest in climate adaptation strategies. Hence, policy should focus on two aspects: (i) increasing farmers’ awareness of climate risks and potential benefits from adopting climate risk adaptation strategies; and (ii) increasing farmers’ capacity for climate risk adaptation by augmenting their assets (e.g., farmland, extension advice, membership in input supply cooperatives, and information on expected rainfall and temperature) while controlling the cost of adaptation. The policy for increasing farmers’ awareness should focus on increasing their access to agricultural extension services. The policy for enhancing farmers’ accessibility to climate risk adaptation strategies should focus on increasing their endowments, for instance, by improving the government provision of extension services, participation in input supply cooperatives, and their access to information on expected rainfall and temperature.

The results of the PSM analysis show that an increasingly positive relationship between climate change adaptation strategies adopted by the surveyed farm households and their food security status. This finding has important policy implications. The Government of Ethiopia and other relevant organizations should encourage farm households to adopt drought-tolerant crop varieties, crop diversification, income diversification, and changing the planting dates as part of their extension strategy to have them adapt to climate change and improve their food security status.

## Figures and Tables

**Table 1.** Definitions, type, and mean values of variables used in the model.

Variables	Definition	Type of variable	Mean

Dependent variables			
Drought-tolerant maize	1 if the household grows drought tolerant maize varieties, 0 otherwise	Dummy	0.60
Income diversification	1 if the household diversifies income sources, 0 otherwise	Dummy	0.45
Crop diversification	1 if the household uses crop diversification strategy, 0 otherwise	Dummy	0.49
Changing planting dates	1 if the household changes planting dates, 0 otherwise	Dummy	0.53
Decision to adapt	1 if the household decides to adopt at least one adaptation strategy, 0 otherwise	Dummy	0.74
Food security	1 if the household is food-secure, 0 otherwise	Dummy	0.60
Independent variables Gender	1 if the household head is male, 0 otherwise	Dummy	0.91
Age	Age of the household head in years	Continuous	48.66
Family size	Number of household members	Continuous	6.88
Experience	Farming experience of the household head in years	Continuous	31.20
Education level	Educational level of the household head in years	Continuous	5.20
Farmland	Total farm size owned by the household in hectares	Continuous	1.92
Extension contact	Number of contacts with extension agent in cropping season	Continuous	2.58
Distance to market	Distance to the main market in km	Continuous	8.07
Distance to agri office	Distance to the agricultural development agent office in km	Continuous	3.05
Membership	1 if the household is a member of an agricultural input supply cooperatives	Dummy	0.40
Demonstration visit	1 if the household visits farm demonstration sites, 0 otherwise	Dummy	0.73
Information	1 if the household regularly receives information on expected rainfall and temperature, 0 otherwise	Dummy	0.61

**Table 2.** Summary of descriptive statistics by adopter and non-adopter farm households.

Variables	Total (N = 516)	Adopters (N = 382)	Non-adopters (N = 134)	Test difference

**Dependent variables (food security status)**				
Food security	0.60 (n = 309)	0.66(n = 252)	0.52(n = 70)	0.14***
Mildly food-insecure	0.11(n = 57)	0.12(n = 46)	0.09(n = 12)	0.03*
Moderately food-insecure	0.22(n = 114)	0.18(n = 69)	0.26(n = 35)	−0.08***
Severely food-insecure	0.07(n = 36)	0.04(n = 15)	0.13(n = 17)	−0.09***
**Independent Variables**				
Gender	0.91(n = 472)	0.90 (n = 343)	0.96 (n = 129)	−0.06
Age	48.66 (0.55)	49.46 (12.87)	48.24 (11.80)	1.22
Family size	6.88 (2.38)	7.10 (2.40)	6.34(2.25)	0.76
Experience	31.20 (13.13)	34.33(13.27)	29.79(12.76)	4.54***
Education level	5.20(3.20)	5.60 (4.90)	5.09(2.21)	0.51
Farmland	1.92(1.06)	2.02(1.09)	1.66(0.95)	0.36***
Extension contact	2.58(2.32)	2.66(2.4)	2.55(2.10)	0.10
Distance to market	8.07(5.58)	7.31(5.89)	8.33(4.50)	−1.02**
Distance to Agri office	3.05(4.69)	3.05(4.46)	3.04(5.30)	0.01
Membership	0.40 (n = 208)	0.45(n = 172)	0.37(n = 50)	0.13***
Demonstration visit	0.73(n = 378)	0.74(n = 283)	0.71(n = 95)	0.03
Information	0.61(n = 313)	0.69(n = 265)	0.36(n = 48)	0.39***

*, **, *** denote the differences are statistically significant at 1%, 5% & 10% significance level, respectively. Standard deviation for continuous variables and number of observations for dummy variables are reported in the parentheses.

**Table 3.** Multivariate probit estimates for the factors associated with the choice of climate risk adaptation.

Variables	Use drought-tolerant crops varieties Coef. Std. Err.		Crop diversification Coef. Std. Err.		Income diversification Coef. Std. Err.		Changing the planting dates Coef. Std. Err.	

Age	0.011**	0.009	−0.014	0.009	−0.29	0.217	0.015	0.009
Sex	0.533	0.225	0.24	0.213	0.015	0.009	−0.099	0.237
Family size	0.016	0.025	0.059**	0.025	0.061**	0.025	0.05*	0.028
Education	0.014	0.017	0.026	0.017	0.023	0.017	0.029	0.019
Experience	0.015*	0.008	0.017**	0.008	0.014	0.009	−0.005	0.009
Farmland	0.029**	0.055	0.08**	0.054	0.035	0.054	0.092	0.06
Extension	0.029	0.026	−0.004	0.025	0.061**	0.028	0.061**	0.032
Membership	0.351***	0.122	0.22**	0.119	0.408***	0.121	0.438***	0.138
Demonstration	0.212	0.134	0.012	0.132	−0.129	0.135	−0.117	0.148
Information	0.673***	0.121	0.562***	0.12	0.308 **	0.12	0.54 ***	0.138
DAgriOffice	−0.006	0.012	−0.013	0.013	0.006	0.014	0.01	0.014
Distance to market	−0.029	0.011	−0.018	0.011	−0.023	0.011	−0.025	0.012
_cons	−0.059	0.430	−0.678	0.419	−0.122	0.421	−2.017	0.47
Rho 2	0.385***	0.063						
Rho 3	0.584***	0.051	0.293***	0.063				
Rho 4	0.132***	0.074	0.47***	0.062	0.036*	0.075		
Number of obs = 516 Wald x2(48) = 147.60 Prob > x2 = 0.000 Log likelihood = −166.649 Likelihood ratio test of rho21 = rho31 = rho41 = rho32 = rho42 = rho43 = 0: x2 (6) = 167.561 Prob > x2 = 0.000								

***, ** and * indicates significant difference at 1%, 5% and 10% probability levels.

**Table 4.** Impacts of the adaptation options on household food security.

Outcome indicators	Matching algorisms	ATT	t-values	Number of treated	Number of control

Food security	NNM	0.023	0.20	368	132
	KBM	0.028	0.40	360	128

Note: NNM represents the nearest neighbor matching and KBM represents the kernel-based matching.

**Table 5.** Indicators of covariate balancing (before and after matching).

Outcome Indicators	Matching algorisms	Median absolute (before matching)	Median absolute bias (after matching)	% bias reduction	Value of R^2^-before matching	Value of R^2^ – after matching	Joint significance of covariates before matching	Joint significance of covariates after matching

Food security	NNM	19.5	6	69.23	0.150	0.018	0.000	0.143
	KM	19.5	6.5	72.01	0.150	0.017	0.000	0.211

Note: NNM stands for the nearest neighbor matching and KM stands for the kernel matching

**Table 6.** Distribution of estimated propensity scores.

Categories	Obs	Mean	Std. Dev.	Min	Max

Total households	516	0.740	0.181	0.200	0.986
Adopter farmers	382	0.785	0.151	0.245	0.986
Non- adopter farmers	134	0.612	0.197	0.201	0.954

## Footnotes

1. Adopter, in this study, is defined as farm households who use at least one climate adaptation strategy to reduce the adverse effect of climate risk.

## Appendix

In this study, the household food security/insecurity were calculated using the Household Food Insecurity Access Prevalence (HFIAP) indicators. Firstly, we coded frequency-of-occurrence as 0 for all cases where the answer to the corresponding occurrence question was “no” (i.e., if the answer to ${\text{Q}}_{1}$ was “no” then frequency-of-occurrence was coded as ${\text{Q}}_{1}=0$ and so on). If the answer to the occurrence question was “yes”, then a frequency-of-occurrence question was coded as 1 for all cases where the situation occurred rarely, 2 for sometimes, and 3 for often. In short, each occurrence question (Table 1–A) was assigned four alternative codes (e.g., ${\text{Q}}_{1}$ was coded as ${\text{Q}}_{1}=0$ for no occurrence, ${\text{Q}}_{1}=1$ for rare occurrence, ${\text{Q}}_{1}=2$ for occasional occurrence, or ${\text{Q}}_{1}=3$ for frequent occurrence). Secondly, the Household Food Insecurity Access (HFIA) category variable was calculated for each household using the assigned codes of the degree of food security/insecurity in which it fell. Accordingly, four categories of food in/security status were created sequentially, (1 = food secure, 2 = mildly food insecure, 3 = moderately food insecure, and 4 = severely food insecure), to ensure that households were classified according to their most severe response.

Each category of the household food security/insecurity was calculated from the above table as:

$$Food\;\text{s}ecure=1\;\text{if}\;[\left(Q_{1}=0\;\text{or}\;Q_{1}=1\right)\;\text{and}\;Q_{2}=0\;\text{and}\;Q_{3}=0\;\text{and}\;Q_{4}=0\;\text{and}\;Q_{5}=0\;\text{and}\;Q_{6}=0\;\text{and}\;Q_{7}=0\;\text{and}\;Q_{8}=0\;\text{and}\;Q_{9}=0]$$

$$Mildly\;food\;insecure=2\;\text{if}\;[\left(Q_{1}=2\;\text{or}\;Q_{1}=3\;\text{or}\;Q_{2}=1\;\text{or}\;Q_{2}=2\;\text{or}\;Q_{2}=3\;\text{or}\;Q_{3}=1\;\text{or}\;Q_{4}=1\right)\;\text{and}\;Q_{5}=0\;\text{and}\;Q_{6}=0\;\text{and}\;Q_{7}=0\;\text{and}\;Q_{8}=0\;\text{and}\;Q_{9}=0]$$

$$Moderately\;food\;insecure=3\;\text{if}\;[\left(Q_{3}=2\;\text{or}\;Q_{3}=3\;\text{or}\;Q_{4}=2\;\text{or}\;Q_{4}=3\;\text{or}\;Q_{5}=1\;\text{or}\;Q_{5}=2\;\text{or}\;Q_{6}=1\;\text{or}\;Q_{6}=2\right)\;\text{and}\;Q_{7}=0\;\text{and}\;Q_{8}=0\;\text{and}\;Q_{9}=0]$$

$$Severely\;food\;insecure=4\;\text{if}\;[Q_{5}=3\;\text{or}\;Q_{6}=3\;\text{or}\;Q_{7}=1\;\text{or}\;Q_{7}=2\;\text{or}\;Q_{7}=3\;\text{or}\;Q_{8}=1\;\text{or}\;Q_{8}=2\;\text{or}\;Q_{8}=3\;\text{or}\;Q_{9}=1\;\text{or}\;Q_{9}=2\;\text{or}\;Q_{9}=3]$$

Finally, the household food security was calculated by dividing the number of households in one category to the total number of households in the four categories.

**Table 1-–A.** Questions in the food security/insecurity

No.	Occurrence Questions

${\text{Q}}_{1}$	In the past four weeks, did you worry that your household would not have enough food?
${\text{Q}}_{2}$	In the past four weeks, were you or any household member not able to eat the kinds of foods you preferred because of a lack of resources?
${\text{Q}}_{3}$	In the past four weeks, did you or any household member have to eat a limited variety of foods due to a lack of resources?
${\text{Q}}_{4}$	In the past four weeks, did you or any household member have to eat some foods that you really did not want to eat because of a lack of resources to obtain other types of food?
${\text{Q}}_{5}$	In the past four weeks, did you or any household member have to eat a smaller meal than you felt you needed because there was not enough food?
${\text{Q}}_{6}$	In the past four weeks, did you or any household member have to eat fewer meals in a day because there was not enough food?
${\text{Q}}_{7}$	In the past four weeks, was there ever no food to eat of any kind in your household because of a lack of resources to get food?
${\text{Q}}_{8}$	In the past four weeks, did you or any household member go to sleep at night hungry because there was not enough food?
${\text{Q}}_{9}$	In the past four weeks, did you or any household member go a whole day and night without eating anything because there was not enough food?
